# Prism adaptation combined with eye movement training for unilateral spatial neglect after stroke: Study protocol for a single-blind prospective, randomized controlled trial

**DOI:** 10.3389/fneur.2022.1081895

**Published:** 2023-01-05

**Authors:** Yu-xuan Yang, Ling-ling Wang, Juan Du, Yao-min Luo, Yu-lei Xie, Bo Zhang, Han Zhang

**Affiliations:** ^1^Department of Rehabilitation Medicine, The Second Clinical Medical School of North Sichuan Medical College, Nanchong Central Hospital, Nanchong, China; ^2^Department of Rehabilitation Medicine, The Affiliated Hospital of North Sichuan Medical College, Nanchong, China; ^3^School of Rehabilitation, China Rehabilitation Research Center, Capital Medical University, Beijing, China

**Keywords:** stroke, unilateral spatial neglect, prism adaptation, eye movement training, combined therapy

## Abstract

**Background:**

Unilateral spatial neglect (USN) is a complex neurological syndrome that often reduces rehabilitation outcomes, prolongs patients' hospital stays, and decreases their quality of life. However, the current therapies for USN have varying efficacy. We will explore a new treatment option that combines prism adaptation (PA) with eye movement training (EMT) for the treatment of USN after stroke.

**Methods:**

We will conduct a single-blind, prospective, randomized controlled trial to assess the efficacy of the combined intervention (PA & EMT) on USN in an inpatient rehabilitation setting. The study aims to recruit 88 patients with USN after an ischemic or hemorrhagic stroke. Participants will be randomly assigned to the following four groups: (1) PA group (*n* = 22), (2) EMT group (*n* = 22), (3) PA and EMT group (*n* = 22), and (4) control group (*n* = 22). All groups will receive 10 sessions of interventions over 2 weeks, 5 times per week. Blinded assessors will conduct a baseline assessment, a post-intervention assessment, and a follow-up assessment (2 weeks post-intervention). The primary outcome measure will use the Behavioral Inattention Test-Conventional Subset (BIT-C) and Catherine Bergego Scale (CBS) to assess the levels of USN. Secondary outcome measures will assess the patient's ability to perform activities of daily living using the Modified Barthel Index (MBI). Patients who completed all treatment and assessment sessions will be included in the final analysis.

**Discussion:**

This study will explore the effects of 10 sessions of combined interventions (PA & EMT) on USN and functional capacity. This study has the potential to identify a new, evidence-based treatment option and provide new ideas for the treatment of USN.

**Ethics and dissemination:**

The study protocol has been approved by the Nanchong Central Hospital. Written informed consent will be obtained from all the participants. The results of this study will be disseminated to the public through scientific conferences and a peer-reviewed journal.

**Trial registration:**

ChiCTR, ChiCTR2100049482. Registered on 2 August 2021, http://www.chictr.org.cn/showproj.aspx?proj=130823.

## 1. Introduction

Stroke is the second cause of death and the leading cause of disability worldwide (1). Poststroke patients usually suffer from multiple dysfunctions and complications that affect their health-related quality of life (2). Unilateral spatial neglect (USN) is a frequent and disabling condition after stroke, affecting approximately 30% of acute and subacute stroke survivors (3). USN is mainly related to damage to neural networks associated with spatial information processing and attentional control (4) and is defined as the inability to orient, detect, or respond to relevant stimuli in the visual field opposite to the brain lesion and unrelated to sensory and motor dysfunction (5). In clinical, approximately 40% of patients with USN are consistently affected by neglect symptoms (6). Compared to other stroke survivors, patients with USN are associated with poorer rehabilitation outcomes of other stroke symptoms (7) and longer hospital stays (8, 9). In addition, USN increases the consumption of health resources and adds to the burden on families (10, 11).

Since the early 1970s, various rehabilitation techniques have been proposed to reduce the disability caused by USN after stroke, including visual scanning training, trunk rotation, optokinetic stimulation, feedback or cueing, virtual reality, repetitive transcranial magnetic stimulation, and prism adaptation (PA) (12, 13). Monotherapy is frequently used in clinical research for USN, but overall, the level of evidence remains low. PA has been a hot research topic in recent years for the treatment of USN, with most studies supporting PA as an effective intervention while other studies were contradictory (14, 15). One possible explanation for the inconsistent results is that USN is a complex neurological syndrome with different manifestations for different neglect types and crossover symptoms between various neglect subtypes (16). Some researchers have suggested that combination therapy may produce more intense and long-lasting effects (17), and combination therapy is also the most frequently investigated USN intervention and shows promise for improving USN symptoms (18). The combination of different treatments may produce greater efficacy through similarities and differences in treatment mechanisms. Based on this, we speculate that combining PA with one approach will yield better results. In previous studies, Saevarsson et al. (19) and Choi et al. (20) combined PA with neck vibration and functional electrical stimulation, respectively, and both showed that the combined intervention better improved USN symptoms. However, the combination of these two studies only increased the number of interventions without mentioning the possible theoretical basis. Barrett et al. (21) inferred from animal models that stroke can induce classic visual–perceptual spatial neglect and motor intention deficits. Choosing a treatment option that intervenes in both areas may be a viable approach.

In this trial, we plan to combine PA and eye-tracking-based eye movement training (EMT) to treat poststroke USN. PA is a “bottom-up” approach (22), and it influences the level of sensory-motor through visuomotor adaptation to reduce symptoms of spatial neglect and, in particular, to improve spatial motor-intentional “aiming” deficits (23). PA was first proposed to treat patients with USN in 1998 (24), and a battery of studies has shown that PA improves not only the performance of patients with USN on neglect assessments (25–27) (e.g., BIT-C, CBS, and bell test) but also on neglect-related processes (15, 28–30). In addition, the sensorimotor after-effects of PA extend to the cognitive domain of patients with USN, for example, in complex spatial cognitive tasks required in daily life (navigation and terrain memory) (31), simple sound source localization abilities (32), etc. EMT is another USN treatment based on the attention disorder doctrine and belongs to the “top-down” approach. Similar to visual scanning training, EMT aims to improve the patient's ability to voluntarily orient his spatial attention toward the neglected side (33) and is characterized by repetitive practice of compensatory visual behaviors. Previous studies have shown that repetitive practice of compensatory visual behaviors can improve USN (34), and Leal Rato, M et al. also showed that eye gaze direction in patients with USN modulates spatial attention and that perception of direct gaze reduces visuospatial deficits in neglected patients (35). Although USN had been classically thought of as a “parietal syndrome” associated with lesions in visuospatial integration at the posterior parietal cortex (36), it has become evident that USN involves a disturbance in the widespread attention network (4), as well as the impact of attention deficits on visuospatial neglect, such as poor sustained attention and attentional shifting disorders (37). Therefore, EMT to improve visuospatial attention may be a treatment for USN, and this technique is still widely used in clinical practice (22).

Our study aims to investigate the efficacy of PA combined with EMT in the treatment of USN. We hypothesized that sequential use of these two interventions would produce a positive synergistic effect of 1 + 1 over 2, resulting in better improvement of USN symptoms in patients with poststroke.

## 2. Methods

This study was confirmed using a checklist in the SPIRIT reporting guidelines (38).

### 2.1. Study design

**Figure 1:**
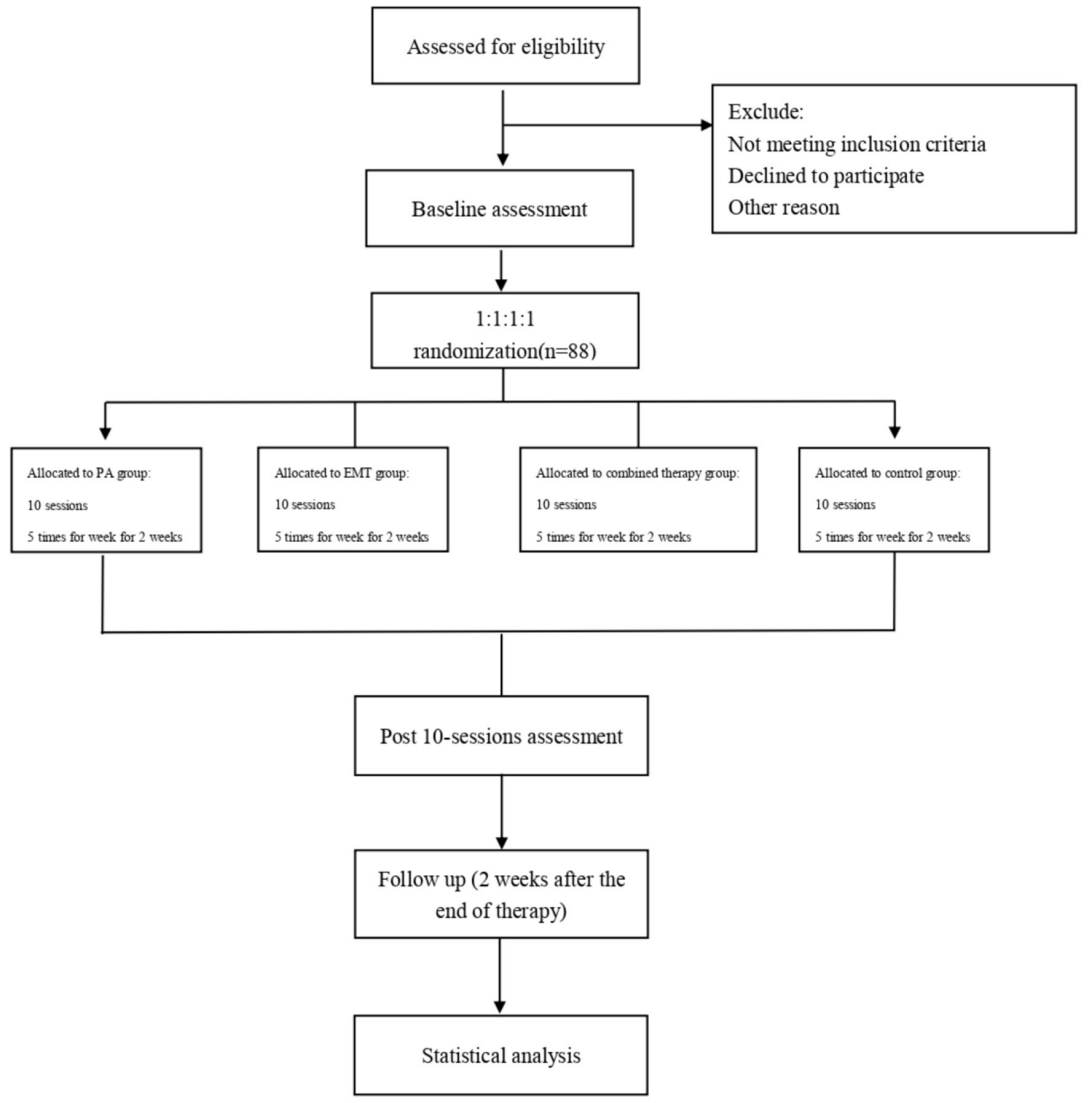
Flowchart of the study design. PA, prism adaptation. EMT, eye movement training.

The study will be conducted as a single-blind, prospective, randomized controlled trial that will be conducted at the Second Clinical Medical College of North Sichuan Medical University. The protocol has been registered with the China Clinical Trial Registry (Item No.: ChiCTR2100049482). Our study will evaluate the effectiveness of EMT combined with PA in patients with poststroke USN, and the findings might provide a rationale for an approach of EMT combined with PA in patients with USN. A total of 72 patients will be recruited for this study and will be randomly assigned to four groups (1:1:1:1). All patients will receive conventional rehabilitation, as well as one of the three types of training: PA, EMT, PA, and EMT. To assess the efficacy, all participants will be assessed at three visits, including baseline, posttreatment, and 2 weeks after the end of treatment. The diagram and schedule for the study are shown in Figure 1 and Table 1.

**Table 1:** Schedule of enrollments, interventions, and assessments.

		**Screening**	**Randomization**	**Intervention**	**Follow up**
**Time point**		**Within one weeks**	**Day 0**	**2 weeks** **(10 sessions)**	**Post-2 weeks**
			**T**_0_	**T**_1_	**T**_2_
Enrollments	Informed consent	√			
	Demographic characteristics	√			
	Medical history	√			
	Eligibility assessment	√			
	Radom allocation		√		
Intervention	Conventional rehabilitation			√	
	PA			√	
	EMT			√	
	PA & EMT			√	
Assessment	Cognitive level (MMSE)	√		√	
	Behavioral inattention test-conventional subset			√	√
	Catherine bergego scale			√	√
	Modified barthel index			√	√

PA, prism adaptation; EMT, eye movement training; MMSE, Mini-mental State Examination.

### 2.2. Consent and eligibility

Potential participants will be primarily screened and those who meet inclusion and exclusion criteria will be invited to participate in this study. All subjects will have an informed consent form signed by themselves or a legal representative prior to undergoing any study procedures. The inclusion and exclusion criteria for selecting participants are listed as follows.

#### 2.2.1. Inclusion criteria

a) Adult patients older than 18 and younger than 80 years.
b) First stroke with ischemic or hemorrhagic brain injury on CT and MRI.
c) The subacute phase of stroke: Duration 1 to 12 weeks after stroke onset.
d) Diagnosis and confirmation of USN: a pathological performance on one subtest of the Behavioral Inattention Test-Conventional Subset (BIT-C).
e) The patient can sit in a stable position.
f) Complete vision or normal after correction.
g) The patient is right-handed.

#### 2.2.2. Exclusion criteria

a) Severe cognitive impairment (MMSE <16) and non-cooperation.
b) Severe USN (star cancellation tests <8).
c) Severe non-spatial attention deficit (digital checking method).
d) Patients with severe organ diseases.
e) Inability to comply with the time frame of this study.
f) Unsigned informed consent.

All subjects will sign an informed consent document before undergoing any study procedures.

### 2.3. Sample and recruitment

Patients with USN in the subacute phase of stroke will be recruited from 2 November 2021 to 1 June 2023 at the Second Clinical Medical College of North Sichuan Medical University. Recruited participants will be required to meet the USN diagnostic criteria, including nurse or family member reports of disproportionate orientation toward the impaired side, and <52 stars were removed from the cancellation test. Patients will be initially screened through a case system or clinician notification and will be carefully evaluated for meeting eligibility criteria once they have signed an informed consent form.

### 2.4. Sample size estimation

In the preexperiment, the changes in BIT-C scores before and after the intervention were 39.33 ± 18.717 for the combined intervention group, 26.33 ± 7.638 for the eye-movement training group, and 20 ± 2.828 for the PA training group. Using the PASS software, the probability α of occurrence of the Type I error was set to 0.05, the probability β of occurrence of the Type II error was calculated as 0.2, the calculation results showed that the combined intervention and eye-movement training groups required 20 samples each, and 9 samples were required for each of the combined intervention and PA training groups. Therefore, taking into account a 10% sample dropout rate, the total sample size was finally determined to be 88 cases, with 22 cases in each group.

### 2.5. Randomization and blinding

A random number list is generated by a computer and consists of 88 random numbers. The random numbers were arranged from the smallest to the largest to obtain the serial number R. It is stipulated that group A (PA) with *R* = 1–22, group B (EMT) with *R* = 23–44, group C (PA & EMT) with *R* = 45–66, and group *D* (control) with R = 67–88. The resulting sequence of random assignments was placed in sequentially coded, sealed, impermeable envelopes. The investigator in charge of recruitment opens the envelope according to the order of patient enrollment and assigns the subjects to the appropriate subject group.

This study is a single-blind design, and only the investigator conducting the assessment is blinded to group assignment. The therapist cannot be blinded due to using the supervised intervention. In addition, blinding of subjects is not feasible due to the difference in intervention methods. All outcome assessments for this study will be conducted by a separate professional therapist who is not involved in any other part of the study. Moreover, participants will be unblinded when any clinical situation associated with adverse events or patient withdrawal occurs.

### 2.6. Interventions

All subjects will receive conventional rehabilitation during the intervention period, as well as appropriate interventions according to the group.

#### 2.6.1. Conventional intervention

Conventional rehabilitation therapy includes physical therapy, occupational therapy, and acupuncture. Physical therapy includes muscle strength and endurance training, joint range of motion training, balance and coordination training, gait training, etc. Occupational therapy includes training in activities of daily living (ADL) (e.g., dressing, eating, brushing teeth, and washing face, etc.). Acupuncture includes acupuncture points such as Baihui, DiCang, Shoulder, Quchi, Hand SanLi, Neiguan, HeGu, LiangQiu, Blood Sea, FengShi, Foot SanLi, YangLingQuan, SanyinJiao, and Taichong.

#### 2.6.2. Prism adaptation

Prism adaptation is a non-invasive, affordable, convenient technique to assess visuomotor plasticity and ameliorate the symptoms of USN (39). During a PA session, the patient wears goggles with prism lenses that induce a deviation of the visual field toward the ipsilesional side of space and perform a series of pointing movements toward a visual target. PA training was performed using a black box with parameters as described by Spaccavento et al. (height = 30 cm, depth = 34 cm at the center and 18 cm at the periphery, and width = 72 cm) (33). The PA process consists of three steps: (1) aiming in the direction of visual targets without goggles to obtain a reference frame (pretest); (2) 90 aiming movements in the direction of visual targets with prisms that deviate from the environment approximately 10**°** to the right or left (prismatic exposure). Initially, the movements are deviated toward the right or left, and then, the subject progressively corrects his errors; (3) aiming toward visual targets without the prisms to measure the after-effects. According to the patient's training performance, the PA training schedule was 15–20 min/session, 1 session/day, and 5 days/week, with a treatment period of 2 weeks.

#### 2.6.3. Eye movement training

**Figure 2:**
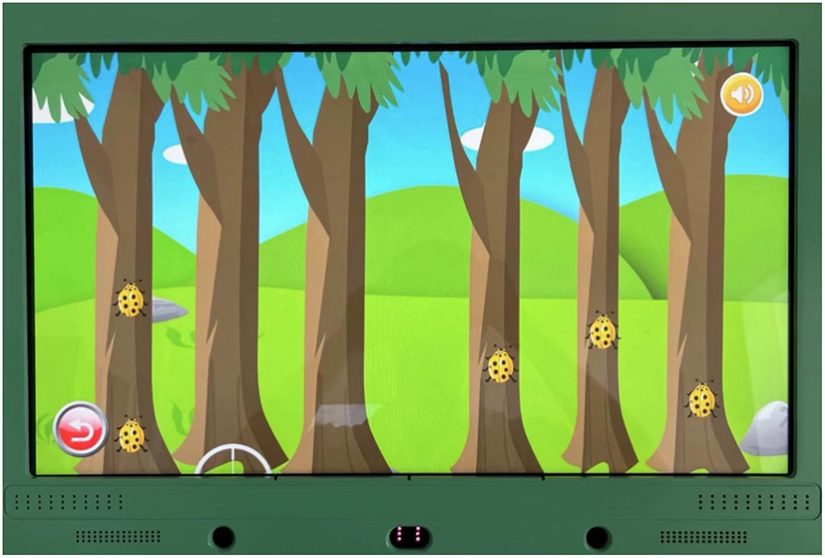
Eye movement training device (image from ourselves).

The eye movement training will be performed based on a high-performance EMT instrument (Figure 2, Hangzhou Jizhi Medical Technology Co., Ltd., Model: JZ-RZ-20US). The insect shoot-down task of the cognitive rehabilitation training and assessment system will be selected as the EMT task (search and gaze). The insect shoot-down task will be set at easy, moderate, and difficult levels (depending on the patient's training performance), left or right field of view (the choice of left or right visual field depends on the patient's side of neglect), during which the insect will randomly present on the left or right side of the screen and move from bottom to top. Under the guidance of the therapist, the patient spontaneously searches for these signs, eliminates them by gazing, and then searches for the next sign until the end of the training. At the same time, this eye-tracking device has an eye-tracking function, which can visually show the patient's eye movement trajectory and facilitate the therapist to better train the patient. The EMT schedule was 15 min/session, 1 session/day, and 5 days/week, with a treatment period of 2 weeks.

### 2.7. Baseline data

Baseline data are collected after informed consent and before randomization. The baseline assessment includes demographic characteristics such as sex, age, time of onset, cause of damage, and assessment scales including the Mini-Mental State Examination, Catherine Bergego Scale, Behavioral Inattention Test-Conventional Subset (BIT-C), and Modified Barthel Index (MBI). All baseline data will be collected *via* paper forms.

### 2.8. Outcome measures

This study will measure outcome indicators at two time points: after the end of the intervention and 2 weeks after the end of the intervention. The measurement of outcome indicators will be conducted by independent therapists. The relevant test nodes can be seen in the study schedule (Table 1).

#### 2.8.1. Mini-mental state examination

The Chinese version of the MMSE was initially developed by Katzman et al. (40) and later widely used in clinical practice and studies. The test includes cognitive assessments in five domains: time and place orientation, memory, attention and calculation, immediate and long-term memory, and language and comprehension. The total score of the test is 30, and the cutoff points for dementia screening are 16/17 for illiterate, 19/20 for those with 1–6 years of education, and 23/24 for those with 7 or more years of education (41).

#### 2.8.2. Primary outcomes

Two main scales are used to assess changes in UNS levels including Catherine Bergego Scale (CBS) and Behavioral Inattention Test-Conventional Subset (BIT-C).

The Catherine Bergego Scale, published by Azouvi et al. in 1996, is an ecologically valid screening tool for spatial neglect with excellent reliability and validity (42). The scale is composed of 10 items and each with a score ranging from 0 (normal) to 3 (severe unilateral neglect). According to the scores, three levels of severe neglect can be distinguished as follows: 1–10 (mild neglect), 11–20 (moderate neglect), and 21–30 (severe neglect).

The behavior inattention test-conventional subset consists of the widely used paper–pencil tests (43): (a) line, letter, and star cancellation tests, (b) figure and shape copying, (c) line bisection, and (d) representative drawing. The sum of scores for each test yields a total BIT-C score, ranging from 0 to 146. The cutoff score for the BIT-C test is 129, and a score below 129 is diagnosed as USN, with lower scores indicating more severe neglect.

(a) In cancellation tests, the signs are presented on an A4 (210 × 297 mm) paper, and the participant was required to respectively cross out all lines, all letters “E” and “R,” and all small stars. There is no time restriction. The number of omitted targets is counted. The maximum scores for these subtests are 36, 40, and 54, respectively.
(b) In the figure and shape copying, the participant has to copy three figures (a four-pointed star, a cube, and a flower) on a sheet of A4 paper, as well as three figures composed of lines. The maximum score is 4.
(c) In the line bisection test, there are three 20 cm horizontal lines on an A4 paper. The participant was asked to search for spatially distributed lines and bisect each line in the middle as accurately as possible. The score ranges from 0 to 3, according to the distance between the mark and the midpoint of each line (0–1 cm 3; 1–2 cm 2; 2–3 cm 1; >3 cm 0). The maximum score is 9.
(d) In the representative drawing, the participant should draw a clock, a human, and a butterfly on an A4 paper based on their memory. The score ranges from 0 to 1 for each drawing, according to symmetry, with a maximum score of 3.

#### 2.8.3. Secondary outcomes

A scale to assess changes in the level of ADL (autonomy) is Modified Barthel Index (MBI).

Modified Barthel Index is a five-level rating scale and evaluates the functional independence and autonomy of the subjects in 10 activities, including (1) bathing, (2) personal grooming, (3) feeding, (4) dressing and undressing, (5) bowel, (6) bladder continence, (7) getting on/off the toilet, (8) stair climbing, (9) moving from wheelchair to bed and return, and (10) walking, with high reliability and stability in people of different sexes and ages (44). The highest score of the MBI is 100, with higher scores indicating increased ADL.

### 2.9. Adverse events

A safety questionnaire will be administered to all participants prior to the administration of the first PA or EMT to reduce the risk of possible symptoms, including dry eyes, headache, and irritability, and will be recorded at the end of each session. Descriptive statistics will be provided for all adverse events.

The following measures will be taken to prevent these events: (a) prior to the intervention, the investigator will communicate adequately to ensure that the patient is in a good state after rest; (b) during the intervention, the investigator will closely monitor the patient's condition and keep records; (c) if the patient feels any discomfort, the intervention will be suspended immediately, the intervention protocol will be adjusted (by increasing the interval of rest), or the intervention will be stopped. If a serious adverse event occurs, we will seek professional evaluation, cover the cost of treatment for the adverse event caused by the trial, and provide some financial compensation.

### 2.10. Dropout criteria

The intervention will be discontinued if the subject meets one or more of the following criteria: (a) the subject has poor compliance and fails to perform the treatment as required, e.g., the subject does not cooperate with the investigator or the subject does not come to treatment on time; (b) medical records are incomplete and affect efficacy or safety evaluation; (c) subject voluntarily withdraws; (d) subject experiences an adverse event (including episodes of ocular pain, headache, and irritability); and (e) the subject has a severely progressive disease or some comorbidity, complications, or specific physiological changes.

Patients who drop out will not be included in the efficacy analysis; if they drop out for reasons such as the occurrence of an adverse event, they will be included in the safety analysis.

### 2.11. Data collection and management

The trial process will be recorded *via* the audio or written form to ensure the authenticity of the intervention. A case report form (CRF) will be used to collect data. Two data managers will enter the data from the CRF into a computer database and cross-check the electronic data for uniformity. All data will be confidential to those outside the study, except for the ethics committee. Experimental data will be used to write clinical research studies. During the course of the study, if subjects discontinue or deviate from the intervention protocol, we will collect as much data as possible for further analysis.

We will use the following methods to facilitate participants' completion of the trial and follow-up: (a) enhance communication between investigators and patients and obtain patients' cooperation whenever possible and (b) provide relevant test results to study patients free of charge.

### 2.12. Data analysis

IBM SPSS Statistics for Windows, Version 26.0 (IBM Corp., Armonk, NY, United States) will be used for statistical analysis. 2-tailed *P* < 0.05 will be considered a statistically significant difference. Continuous variables will be expressed as mean with standard deviation or median with interquartile range, whereas categorical data will be expressed as counts and percentages. Baseline comparisons will be used to examine potential differences between 4 groups. Age, time of onset, and MMSE will be analyzed by ANOVA or Kruskal-Wallis test. Sex, cause of damage will be measured using Chi-square tests. The Kolmogorov-Smirnov test will be used to evaluate the normality of distributions. If a normal distribution is confirmed, one-way ANOVA will be used to examine the effectiveness of intervention between the 4 groups at T1, T2, with Bonferroni correction for multiple comparisons as a *post hoc* test. Otherwise, Kruskal-Wallis (non-parametric test) will be used.

## 3. Discussion

Unilateral spatial neglect is a complex neurological syndrome with a high prevalence and adverse effects. In this study, we design a random and comparison clinical trial to observe the effectiveness of PA and EMT and combined therapy for USN of patients with poststroke.

A major consideration of this study is based on the theoretical model of Barrett et al. (21), who mentioned that stroke-induced unilateral spatial neglect can be characterized by visual–perceptual spatial neglect and motor intention deficits. Many studies have investigated the effectiveness of PA for USN, indicating that PA is a promising intervention to alleviate symptoms of neglect and improve functional outcomes. However, some contrary studies have shown that patients improved only motor-intentional deficits after PA intervention (45, 46). EMT is another effective intervention used in the study. Balslev and Odoj (47) supported the coupling of attention and gaze and argued that interventions on target gaze signals can alleviate visual–perceptual spatial neglect. Therefore, we hypothesized that the combination of the two interventions might both treat the patients' classic visual–perceptual spatial neglect and motor intention deficits, resulting in a more positive and comprehensive effect.

In contrast, although previous studies have explored PA in combination with other treatments, most studies have selected therapies with the same bottom-up approach (19, 20, 48). It is notable that the two interventions chosen for the present study stem from the following two different approaches: the “top-down” approach aims to improve perceptual and behavioral biases by acting on disrupted consciousness and thus on higher-level cognitive processes, and the “bottom-up” approach is a physiological approach that aims to influence sensory-motor levels through passive sensory manipulation or visuomotor adaptation. PA belongs to a bottom-up intervention approach, while EMT belongs to a top-down intervention approach. PA may ameliorate neglect symptoms by improving patients' spatial cognitive processes: recalibration and spatial alignment (49, 50), and imaging studies have shown that PA activates the parietal cortex and cerebellum associated with recalibration and spatial rearrangement (39, 51, 52), as well as altering the balance of activity in bilateral parietal, frontal, and temporal regions (53), and altering frontal-parietal, parietal-temporal, and cerebellar-parietal-hippocampal network connections in the resting state (54, 55). EMT improves spontaneous eye exploration and spatial attention to the space contralateral to the brain injury. An fMRI study showed that EMT induced alterations in brain activation in the striate and extrastriate cortex as well as in oculomotor areas (56). The two showed more differences in neural mechanisms, so the combined intervention of these two approaches may affect the broader brain network associated with USN. Based on this, we chose to combine these two approaches in the present study, which we hypothesized would have positive effects.

Since patients with USN themselves suffer from attention deficits and other cognitive dysfunctions, an unreasonable combination of therapies rather leads to an aggravation of neglect symptoms (48), and therefore, the selection of the combination of different interventions needs to take into account the relevant influencing factors and the patient's tolerance. Saevarsson et al. (19) and Choi et al. (20) combined an active engagement (PA) with a passively received (neck vibration or FES) intervention, both of which showed better efficacy of the combined intervention, but both interventions used in our trial required patients to actively participate, so this may be a limitation of this interventional approach. However, the few patients who completed the intervention described that they were able to accept the intensity of the training and did not experience any particular fatigue or difficulty accomplishing it.

There are other limitations to our study. (a) Our target population was set to patients with subacute stroke, and the efficacy of patients in the chronic phase was not discussed. (b) The efficacy of interventions with active engagement is influenced by cognitive level, and we only mentioned the ability to cooperate with the therapist in the eligibility criteria, discussed the overall efficacy, and did not stratify the analysis of the efficacy of patients with different cognitive levels. (c) Patients with severe USN were excluded from the study, so the efficacy for this group is not yet clear. (d) This is a preliminary exploratory study, and the follow-up time in this trial is only 2 weeks after the end of treatment.

We aimed to conduct a randomized controlled trial to investigate whether the PA combined with EMT has the potential to be a promising treatment option for poststroke USN. If this study provides positive results, it will be possible to recommend that these techniques be implemented in treatment protocols for patients with USN.

## Trial status

This publication is based on version 1 of the PA combined eye movement protocol dated 2 August 2021. The official start of recruitment was on 2 November 2021. The estimated end date of the trial is 1 June 2023, and the recruitment of patients is ongoing.

## Ethics statement

The studies involving human participants were reviewed and approved by the Medical Ethics Committee of Nanchong Central Hospital. The patients/participants provided their written informed consent to participate in this study.

## Author contributions

Y-xY conceptualized and wrote the study. L-lW and Y-lX prepared the manuscript and contributed to the study design. JD provided statistical expertise in clinical trial design. Y-mL is responsible for the assessment of the trial. BZ and HZ reviewed and approved the manuscript for final submission. All authors contributed to the refinement of the study protocol and approved the final manuscript.

## References

[1] Collaborators GBDS. Global, regional, and national burden of stroke and its risk factors, 1990-2019: a systematic analysis for the Global Burden of Disease Study 2019. Lancet Neurol. (2021) 20:795–820. 10.1016/S1474-4422(21)00252-0 34487721 PMC8443449

[2] Bejot Y Bailly H Durier J Giroud M. Epidemiology of stroke in Europe and trends for the 21st century. Presse Med. (2016) 45:e391–e8. 10.1016/j.lpm.2016.10.003 27816343

[3] Esposito E Shekhtman G Chen P. Prevalence of spatial neglect post-stroke: A systematic review. Ann Phys Rehabil Med. (2021) 64:101459. 10.1016/j.rehab.2020.10.010 33246185

[4] Corbetta M Shulman GL. Spatial neglect and attention networks. Annu Rev Neurosci. (2011) 34:569–99. 10.1146/annurev-neuro-061010-113731 21692662 PMC3790661

[5] Heilman KM Valenstein E Watson RT. Neglect and related disorders. Semin Neurol. (2000) 20:463–70. 10.1055/s-2000-13179 11149702

[6] Nijboer TC Kollen BJ Kwakkel G. Time course of visuospatial neglect early after stroke: a longitudinal cohort study. Cortex. (2013) 49:2021–7. 10.1016/j.cortex.2012.11.006 23332473

[7] Di Monaco M Schintu S Dotta M Barba S Tappero R Gindri P. Severity of unilateral spatial neglect is an independent predictor of functional outcome after acute inpatient rehabilitation in individuals with right hemispheric stroke. Arch Phys Med Rehabil. (2011) 92:1250–6. 10.1016/j.apmr.2011.03.018 21807144

[8] Chen P Hreha K Kong Y Barrett AM. Impact of spatial neglect on stroke rehabilitation: evidence from the setting of an inpatient rehabilitation facility. Arch Phys Med Rehabil. (2015) 96:1458–66. 10.1016/j.apmr.2015.03.019 25862254 PMC4519421

[9] Tarvonen-Schroder S Niemi T Koivisto M. Comparison of functional recovery and outcome at discharge from subacute inpatient rehabilitation in patients with right or left stroke with and without contralateral spatial neglect. J Rehabil Med. (2020) 52:jrm00071. 10.2340/16501977-2698 32488283

[10] Buxbaum LJ Ferraro MK Veramonti T Farne A Whyte J Ladavas E . Hemispatial neglect: Subtypes, neuroanatomy, and disability. Neurology. (2004) 62:749–56. 10.1212/01.WNL.0000113730.73031.F4 15007125

[11] Chen P Fyffe DC Hreha K. Informal caregivers' burden and stress in caring for stroke survivors with spatial neglect: an exploratory mixed-method study. Top Stroke Rehabil. (2017) 24:24–33. 10.1080/10749357.2016.1186373 27216085

[12] Teasell R Salbach NM Foley N Mountain A Cameron JI Jong A . Canadian stroke best practice recommendations: rehabilitation, recovery, and community participation following stroke. Part One: Rehabilitation and Recovery Following Stroke; 6th Edition Update 2019. Int J Stroke. (2020) 15:763–88. 10.1177/1747493019897843 31983296

[13] Luaute J Halligan P Rode G Rossetti Y Boisson D. Visuo-spatial neglect: a systematic review of current interventions and their effectiveness. Neurosci Biobehav Rev. (2006) 30:961–82. 10.1016/j.neubiorev.2006.03.001 16647754

[14] Ten Brink AF Visser-Meily JMA Schut MJ Kouwenhoven M Eijsackers ALH Nijboer TCW. Prism adaptation in rehabilitation? No additional effects of prism adaptation on neglect recovery in the subacute phase poststroke: a randomized controlled trial. Neurorehabil Neural Repair. (2017) 31:1017–28. 10.1177/1545968317744277 29192535

[15] Mizuno K Tsujimoto K Tsuji T. Effect of prism adaptation therapy on the activities of daily living and awareness for spatial neglect: a secondary analysis of the randomized, controlled trial. Brain Sci. (2021) 11:347. 10.3390/brainsci11030347 33803412 PMC8001351

[16] Zhang R G He C X Wang D Huang Y K Wang F Y Yang Y H. Progress of visual-motor stimulation intervention for unilateral neglect after stroke. China Rehabilitation. (2021) 36:495–8.

[17] Saevarsson S Halsband U Kristjansson A. Designing rehabilitation programs for neglect: could 2 be more than 1 + 1? Appl Neuropsychol. (2011) 18:95–106. 10.1080/09084282.2010.547774 21660761 PMC4544767

[18] Umeonwuka C Roos R Ntsiea V. Current trends in the treatment of patients with post-stroke unilateral spatial neglect: a scoping review. Disabil Rehabil. (2020) 44:1–28. 10.1080/09638288.2020.1824026 32976719

[19] Saevarsson S Kristjansson A Halsband U. Strength in numbers: combining neck vibration and prism adaptation produces additive therapeutic effects in unilateral neglect. Neuropsychol Rehabil. (2010) 20:704–24. 10.1080/09602011003737087 20503132 PMC3129649

[20] Choi HS Kim DJ Yang YA. The Effect of a Complex Intervention Program for Unilateral Neglect in Patients with Acute-Phase Stroke: A Randomized Controlled Trial. Osong Public Health Res Perspect. (2019) 10:265–73. 10.24171/j.phrp.2019.10.5.02 31673487 PMC6816354

[21] Barrett AM Goedert KM Basso JC. Prism adaptation for spatial neglect after stroke: translational practice gaps. Nat Rev Neurol. (2012) 8:567–77. 10.1038/nrneurol.2012.170 22926312 PMC3566983

[22] Azouvi P Jacquin-Courtois S Luaute J. Rehabilitation of unilateral neglect: Evidence-based medicine. Ann Phys Rehabil Med. (2017) 60:191–7. 10.1016/j.rehab.2016.10.006 27986428

[23] Gammeri R Iacono C Ricci R Salatino A. Unilateral spatial neglect after stroke: current insights. Neuropsychiatr Dis Treat. (2020) 16:131–52. 10.2147/NDT.S171461 32021206 PMC6959493

[24] Rossetti Y Rode G Pisella L Farne A Li L Boisson D . Prism adaptation to a rightward optical deviation rehabilitates left hemispatial neglect. Nature. (1998) 395:166–9. 10.1038/25988 9744273

[25] Facchin A Figliano G Daini R. Prism adaptation and optokinetic stimulation comparison in the rehabilitation of unilateral spatial neglect. Brain Sci. (2021) 11:1488. 10.3390/brainsci11111488 34827487 PMC8615435

[26] Mizuno K Tsuji T Takebayashi T Fujiwara T Hase K Liu M. Prism adaptation therapy enhances rehabilitation of stroke patients with unilateral spatial neglect: a randomized, controlled trial. Neurorehabil Neural Repair. (2011) 25:711–20. 10.1177/1545968311407516 21700922

[27] Fortis P Ronchi R Velardo V Calzolari E Banco E Algeri L . A home-based prism adaptation training for neglect patients. Cortex. (2020) 122:61–80. 10.1016/j.cortex.2018.09.001 30314612

[28] Anelli F Avanzi S Damora A Mancuso M Frassinetti F. Mental time travel and functional daily life activities in neglect patients: Recovery effects of rehabilitation by prism adaptation. Cortex. (2019) 113:141–55. 10.1016/j.cortex.2018.12.003 30660953

[29] Champod AS Frank RC Taylor K Eskes GA. The effects of prism adaptation on daily life activities in patients with visuospatial neglect: a systematic review. Neuropsychol Rehabil. (2018) 28:491–514. 10.1080/09602011.2016.1182032 27181587

[30] Zhu HF Zhang J Wu QF. Study on the improvement effect of prismatic adaptation technique on unilateral spatial neglect after stroke. Hebei Med. (2017) 39:2235–7.

[31] Glize B Lunven M Rossetti Y Revol P Jacquin-Courtois S Klinger E . Improvement of Navigation and Representation in Virtual Reality after Prism Adaptation in Neglect Patients. Front Psychol. (2017) 8:2019. 10.3389/fpsyg.2017.02019 29209253 PMC5701812

[32] Matsuo T Moriuchi T Iso N Hasegawa T Miyata H Maruta M . Effects of prism adaptation on auditory spatial attention in patients with left unilateral spatial neglect: a non-randomized pilot trial. Int J Rehabil Res. (2020) 43:228–34. 10.1097/MRR.0000000000000413 32776764

[33] Spaccavento S Cellamare F Cafforio E Loverre A Craca A. Efficacy of visual-scanning training and prism adaptation for neglect rehabilitation. Appl Neuropsychol Adult. (2016) 23:313–21. 10.1080/23279095.2015.1038386 26583597

[34] Metzler MJ Maiani M Jamieson B Dukelow SP. Clinical provision of compensatory visual training after neurological injury: example of a multisite outpatient program. Disabil Rehabil. (2021) 43:118–25. 10.1080/09638288.2019.1616835 31120310

[35] Leal Rato M Mares I Aguiar de Sousa D Senju A Martins IP. Direct gaze partially overcomes hemispatial neglect and captures spatial attention. Front Psychol. (2018) 9:2702. 10.3389/fpsyg.2018.02702 30697179 PMC6340963

[36] Driver J Mattingley JB. Parietal neglect and visual awareness. Nat Neurosci. (1998) 1:17–22. 10.1038/217 10195103

[37] Rengachary J He BJ Shulman GL Corbetta M. A behavioral analysis of spatial neglect and its recovery after stroke. Front Hum Neurosci. (2011) 5:29. 10.3389/fnhum.2011.00029 21519374 PMC3075878

[38] Chan AW Tetzlaff JM Gotzsche PC Altman DG Mann H Berlin JA . SPIRIT 2013 explanation and elaboration: guidance for protocols of clinical trials. BMJ. (2013) 346:e7586. 10.1136/bmj.e7586 23303884 PMC3541470

[39] Panico F Rossetti Y Trojano L. On the mechanisms underlying Prism Adaptation: A review of neuro-imaging and neuro-stimulation studies. Cortex. (2020) 123:57–71. 10.1016/j.cortex.2019.10.003 31759324

[40] Katzman R Zhang MY Ouang Ya Q Wang ZY Liu WT Yu E . A Chinese version of the Mini-Mental State Examination; impact of illiteracy in a Shanghai dementia survey. J Clin Epidemiol. (1988) 41:971–8. 10.1016/0895-4356(88)90034-0 3193141

[41] Li H Jia J Yang Z. Mini-Mental State Examination in Elderly Chinese: A Population-Based Normative Study. J Alzheimers Dis. (2016) 53:487–96. 10.3233/JAD-160119 27163822

[42] Azouvi P Samuel C Louis-Dreyfus A Bernati T Bartolomeo P Beis JM . Sensitivity of clinical and behavioural tests of spatial neglect after right hemisphere stroke. J Neurol Neurosurg Psychiatry. (2002) 73:160–6. 10.1136/jnnp.73.2.160 12122175 PMC1737990

[43] Wilson B Cockburn J Halligan P. Behavioural Inattention Test; Manual, Fareham. (1987).

[44] Yang H Chen Y Wang J Wei H Chen Y Jin J. Activities of daily living measurement after ischemic stroke: Rasch analysis of the modified Barthel Index. Medicine (Baltimore). (2021) 100:e24926. 10.1097/MD.0000000000024926 33655956 PMC7939171

[45] Striemer CL Danckert J. Dissociating perceptual and motor effects of prism adaptation in neglect. Neuroreport. (2010) 21:436–41. 10.1097/WNR.0b013e328338592f 20220540

[46] Fortis P Chen P Goedert KM Barrett AM. Effects of prism adaptation on motor-intentional spatial bias in neglect. Neuroreport. (2011) 22:700–5. 10.1097/WNR.0b013e32834a3e20 21817924 PMC3165096

[47] Balslev D Odoj B. Distorted gaze direction input to attentional priority map in spatial neglect. Neuropsychologia. (2019) 131:119–28. 10.1016/j.neuropsychologia.2019.05.017 31128129 PMC6667735

[48] Keller I Lefin-Rank G Losch J Kerkhoff G. Combination of pursuit eye movement training with prism adaptation and arm movements in neglect therapy: a pilot study. Neurorehabil Neural Repair. (2009) 23:58–66. 10.1177/1545968308317438 18801912

[49] Redding GM Wallace B. Strategic calibration and spatial alignment: a model from prism adaptation. J Mot Behav. (2002) 34:126–38. 10.1080/00222890209601935 12057886

[50] Redding GM Rossetti Y Wallace B. Applications of prism adaptation: a tutorial in theory and method. Neurosci Biobehav Rev. (2005) 29:431–44. 10.1016/j.neubiorev.2004.12.004 15820548

[51] Kuper M Wunnemann MJ Thurling M Stefanescu RM Maderwald S Elles HG . Activation of the cerebellar cortex and the dentate nucleus in a prism adaptation fMRI study. Hum Brain Mapp. (2014) 35:1574–86. 10.1002/hbm.22274 23568448 PMC6869654

[52] Terruzzi S Crivelli D Pisoni A Mattavelli G Romero Lauro LJ Bolognini N . The role of the right posterior parietal cortex in prism adaptation and its aftereffects. Neuropsychologia. (2021) 150:107672. 10.1016/j.neuropsychologia.2020.107672 33188788

[53] Crottaz-Herbette S Fornari E Notter MP Bindschaedler C Manzoni L Clarke S. Reshaping the brain after stroke: The effect of prismatic adaptation in patients with right brain dam age. Neuropsychologia. (2017) 104:54–63. 10.1016/j.neuropsychologia.2017.08.005 28782545

[54] Tsujimoto K Mizuno K Nishida D Tahara M Yamada E Shindo S . Prism adaptation changes resting-state functional connectivity in the dorsal stream of visual attention networks in healthy adults: A fMRI study. Cortex. (2019) 119:594–605. 10.1016/j.cortex.2018.10.018 30471844

[55] Schintu S Freedberg M Gotts SJ Cunningham CA Alam ZM Shomstein S . Prism adaptation modulates connectivity of the intraparietal sulcus with multiple brain networks. Cereb Cortex. (2020) 30:4747–58. 10.1093/cercor/bhaa032 32313949 PMC7526755

[56] Nelles G Pscherer A de Greiff A Forsting M Gerhard H Esser J . Eye-movement training-induced plasticity in patients with post-stroke hemianopia. J Neurol. (2009) 256:726–33. 10.1007/s00415-009-5005-x 19240963

